# miR-24 Regulates Apoptosis by Targeting the Open Reading Frame (ORF) Region of FAF1 in Cancer Cells

**DOI:** 10.1371/journal.pone.0009429

**Published:** 2010-02-25

**Authors:** Wenming Qin, Yi Shi, Botao Zhao, Chengguo Yao, Li Jin, Jiexian Ma, Youxin Jin

**Affiliations:** 1 State Key Laboratory of Molecular Biology, Shanghai Institutes for Biological Sciences, Chinese Academy of Sciences, Shanghai, China; 2 St Luke's Hospital, Shanghai, China; Health Canada, Canada

## Abstract

**Background:**

microRNAs (miRNAs) are small noncoding RNAs that regulate cognate mRNAs at the post-transcriptional stage. Several studies have shown that miRNAs modulate gene expression in mammalian cells by base pairing to complementary sites in the 3′-untranslated region (3′-UTR) of the target mRNAs.

**Methodology/Principal Findings:**

In the present study, miR-24 was found to target fas associated factor 1(FAF1) by binding to its amino acid coding sequence (CDS) region, thereby regulating apoptosis in DU-145 cells. This result supports an augmented model whereby animal miRNAs can exercise their effects through binding to the CDS region of the target mRNA. Transfection of miR-24 antisense oligonucleotide (miR-24-ASO) also induced apoptosis in HGC-27, MGC-803 and HeLa cells.

**Conclusions/Significance:**

We found that miR-24 regulates apoptosis by targeting FAF1 in cancer cells. These findings suggest that miR-24 could be an effective drug target for treatment of hormone-insensitive prostate cancer or other types of cancers. Future work may further develop miR-24 for therapeutic applications in cancer biology.

## Introduction

microRNAs (miRNAs) are endogenous, evolutionarily conserved small (approximately 22 nt) noncoding RNAs that have been shown to regulate gene expression post-transcriptionally [1], [2]. miRNAs usually regulate the expression of their target genes by degrading target mRNA transcripts or inhibiting mRNA translation [3], [4]. miRNAs have been implicated in various biological processes including developmental timing, patterning and embryogenesis, differentiation and organogenesis, immune response, growth control and apoptosis. However, the target genes and functions of most miRNAs are still unclear [5]–[9].

Previous studies have shown that miRNAs modulate gene expression in mammalian cells by base pairing to complementary sites in the 3′-untranslated region (3′-UTR) of their target mRNAs [4], [10], [11]. More recently, it has been demonstrated that miRNAs can regulate target mRNAs by binding to the amino acid coding sequence (CDS) [12]–[14].

Apoptosis is a form of programmed cell death (PCD) that occurs in multicellular organisms. Certain types of damage trigger a series of biochemical events, leading to a characteristic cell morphology and death [15], [16]. It is a well-orchestrated cellular mechanism that balances the effects of cell proliferation and cell death[17]. It seems clear that the tight regulation of apoptotic function through miRNAs is critical in development and other cellular processes. Several miRNAs that regulate apoptosis have been identified, though none of them regulate apoptosis by binding the CDS region of their targets [17]–[22]. Recently, it has been shown that miRNAs may act as oncogenes and tumor suppressors by targeting key regulators of cell growth [23]–[25].

A novel class of chemically engineered oligonucleotides, termed ‘antagomirs’, can effectively silence endogenous miRNAs and have been used to abolish the aberrant expression of onco-miRNAs [26], [27]. This demonstrates a potential therapeutic application for effective silencing of onco-miRNAs in the treatment of some types of cancer[9].

Fas-associated factor 1 (FAF1) is a component of the death-inducing signaling complex (DISC) and interacts with caspase-8 and FADD although it lacks the “death motifs” typical of apoptosis proteins [28]. Several recent studies showed that over-expression of FAF1 stimulated apoptosis in Jurkat cells, L cells and BOSC23 cells despite the absence of any extrinsic death signal [28]–[30]. FAF1 has been shown to play an important role in normal development and neuronal cell survival, whereas FAF1 downregulation may contribute to multiple aspects of tumorigenesis [31]. Using small interfering RNA (siRNA) to target FAF1, it was observed that down-regulation of FAF1 enhanced the susceptibility to apoptosis triggered by CP [32].

Thus far, it is not known whether FAF1 is regulated by miRNAs. To our knowledge, no study has investigated how miRNAs that target the CDS region of the target gene contribute to tumor apoptosis. In this study, we show that miR-24 can regulate human FAF1 (hFAF1) expression through binding to the CDS region of hFAF1 mRNA. Moreover, we demonstrate that miR-24 regulates apoptosis and proliferation in DU-145, HGC-27, MGC-803 and HeLa cells by targeting the FAF1 gene. These findings suggest that miR-24 could be a potential drug target gene for the treatment of hormone-insensitive prostate cancer and other types of cancers.

## Results

### miR-24-ASO-Mediated Down-Regulation of miR-24 Induces Apoptosis and Affects Proliferation in DU-145 Cells

**Figure 1:**
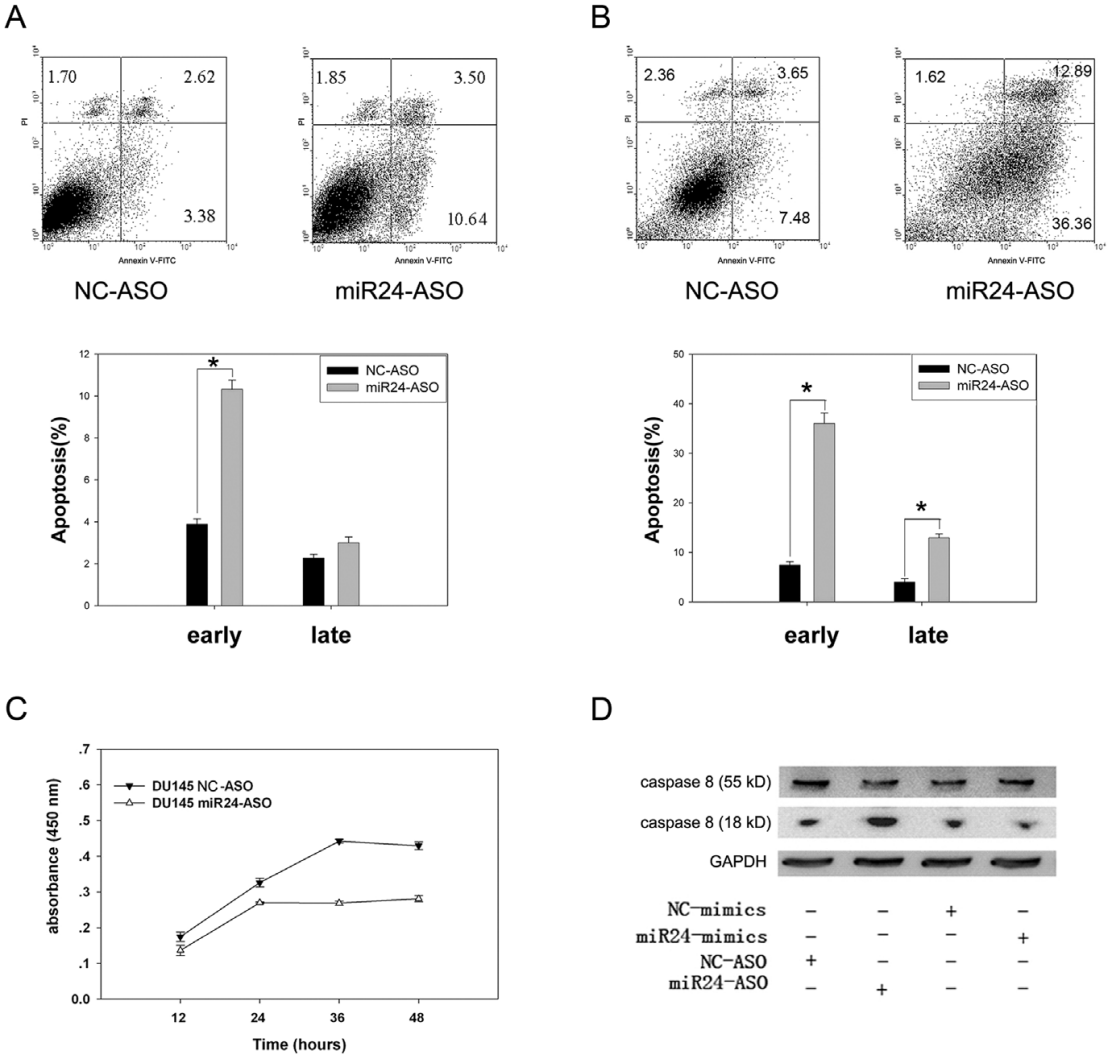
Down-regulation of miR-24 induces apoptosis and affects proliferation in DU-145 cells. (A) DU-145 cells were treated with 20 nM miR-24-ASO or NC-ASO for 48 h. Apoptosis was measured by FACS with Annexin V and propidium iodide staining (n = 3±SE; *p<0.01). Down-regulation of miR-24 induced apoptosis in DU-145 cells. (B) Apoptosis was induced for 2 h with 1 µM staurosporine, 48 h after transfection 20 nM miR-24-ASO or NC-ASO (n = 3±SE; *p<0.01). Apoptosis sensitivity increased significantly after transfection with miR-24-ASO. (C) Cell proliferation was measured by CCK-8 kit at the indicated time points after transfection with 20 nM miR-24-ASO or NC-ASO (n = 3±SE). Down-regulation of miR-24 affected the proliferation of DU-145 cells. (D) Caspase-8 activation was detected by western blot analysis of pro-caspase 8 and the mature form of caspase-8 after 24 h of treatment (as above). Caspase-8 was activated after transfection with miR-24-ASO.

To explore the functional effect of miR-24 on cell survival, we measured apoptosis in DU-145 cells transfected with miR-24-ASO. Down-regulation of miR-24 by miR-24-ASO increased apoptosis in DU-145 cells, as measured by FACS analysis of cells for propidium iodide and annexin V staining (Fig. 1A). Staurosporine was used to induce apoptosis at 48 hours after miR-24-ASO transfection. Apoptosis was much higher in DU-145 cells transfected with miR-24-ASO than in cells transfected with Negative Control NC-ASO (Fig. 1B). Next, the effect of miR-24 on the proliferation of DU-145 cells was explored with a CCK8 counting kit. As predicted, the proliferation of DU-145 cells was greatly reduced after miR-24 was down-regulated by miR-24-ASO, possibly because of apoptosis induced by miR-24-ASO (Fig. 1C). The expression of caspase-8 was also elevated in cells transfected with miR-24-ASO, while caspase-8 was slightly protected from activation in cells over-expressing miR-24 (Fig. 1D). These data suggest that miR-24 down-regulation in DU-145 cells can induce apoptosis, possibly through the caspase-8 pathway.

### miR-24 Regulates the FAF1 Gene by Binding to the ORF Region of FAF1

**Figure 2:**
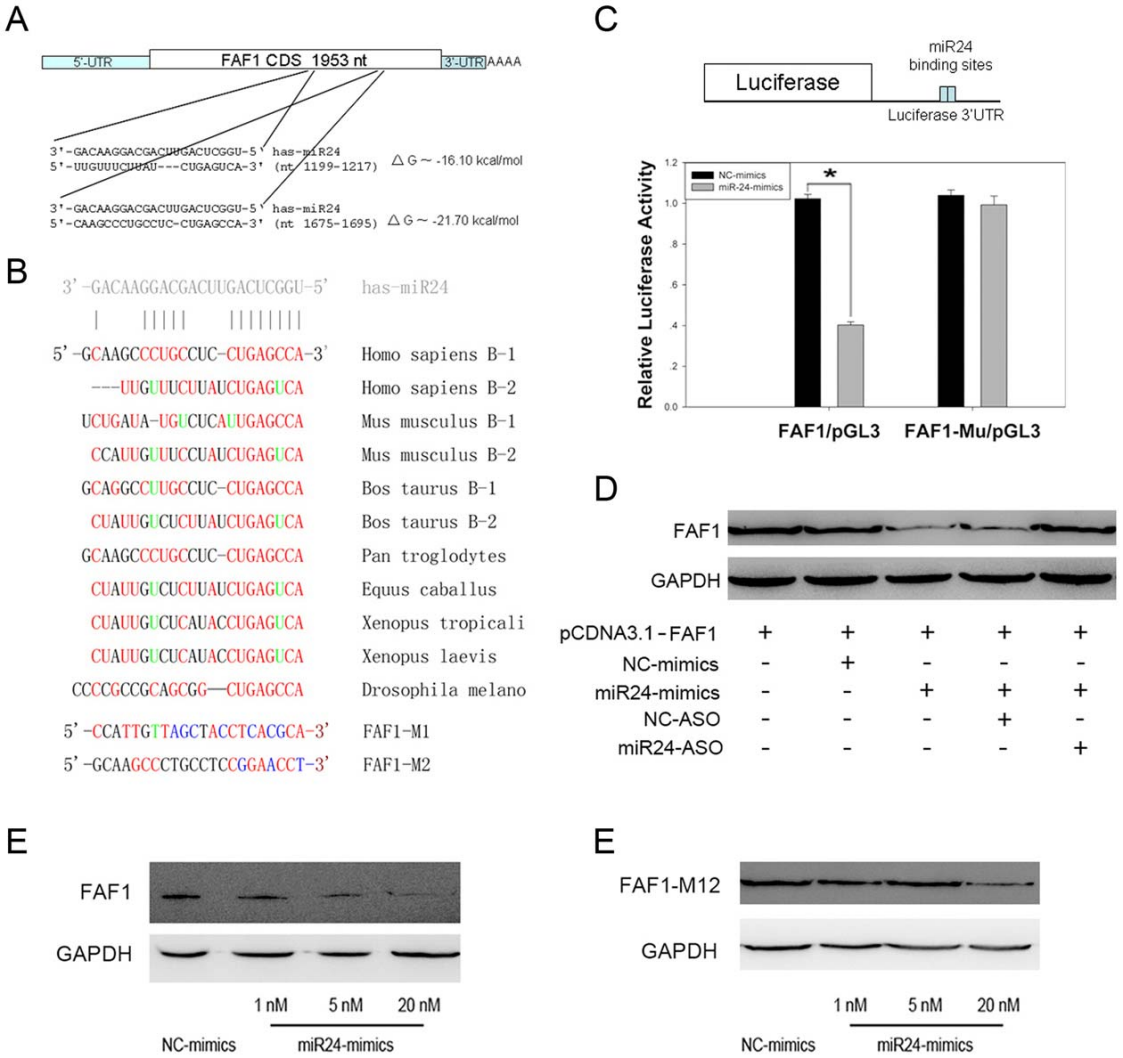
miR-24 regulates FAF1 by targeting its ORF region. (A) RNA22 software was used to predict that the CDS region of FAF1 mRNA harbors two putative miR-24 binding sites. The sequence of miR-24, its binding sites, and energies are shown. (B) The predicted binding sites of FAF1 were conserved in eight different species. The bases altered in the mutant FAF1 constructs are also shown. Red: paired bases; green: G:U pair; blue: mutant bases. (C) 293T cells were transfected with a reporter vector consisting of a luciferase gene containing the wild type or mutated miR-24 binding sites) in its 3′-UTR region (FAF1/pGL3 or FAF1-M12/pGL3, 20 ng/well in each 24-well plate). The cells were also transfected with a CMV-Renilla luciferase vector (20 ng/well in each 24-well plate) as an internal standard. Expression of the luciferase containing predicted miR-24 binding sequences was decreased by treatment with 20 nM miR-24-mimic compared to treatment with NC-mimic, while the luciferase containing mutated sequences could not be down-regulated (n = 3±SE; *p<0.01). (D) miR-24 regulates the FAF1 gene. 293T cells were treated as indicated and transfected with 100 ng pcDNA3.1-FAF1. The expression of FAF1 was down-regulated after transfection with 20 nM miR-24-mimic and rescued after 100 nM miR-24-ASO being transfected. (E) Dose-dependent suppression of FAF1 by miR-24 in 293T cells. 293T cells were co-transfected with 100 ng pcDNA3.1-FAF1 and 1, 5, or 20 nM miR-24-mimic after 24 h and immunoblotted as above. (F) Mutant FAF1 could not be down-regulated upon transfection with 1 nM or 5 nM of miR-24-mimic for 24 h. FAF1 was down-regulated slightly after transfection with 20 nM miR-24-mimic; this might because the mutation to FAF1 was somewhat small.

Using methods described by Karginov and colleagues [33] with some modifications, we found that FAF1 is a putative target of miR-24. Analysis with RNA22 software (IBM) indicated that there is no miRNA binding site in the 3′UTR region of the FAF1 gene, but two putative miR-24 binding sites were found within the open reading frame (ORF) region of FAF1 (Fig. 2A). Furthermore, the miR-24 target sequences, especially the ‘seed region’, at the ORF region of FAF1 were found to be highly conserved among eight species (Fig. 2B).

To test whether miR-24 could regulate FAF1 by binding to these two predicted sites, fragments containing these two sequences or mutated sequences were cloned into the 3′UTR region of the luciferase gene of the reporter vector pGL3, named FAF1/pGL3 or FAF1-M12/pGL3 (Fig. 2C). These luciferase reporter vectors containing miR-24 response elements or mutated sequences were co-transfected into 293T cells with an NC-mimic control or miR-24-mimic. Subsequently, the luciferase and Renilla activity in each well was measured. It was found that miR-24 was able to decrease the luciferase activity of the reporter vector containing miR-24 response elements, while the reporter containing mutated sequences was not down-regulated (Fig. 2C). These data show that miR-24 can down-regulate its targets by binding to these two predicted binding sites.

To test whether miR-24 decreases the expression of FAF1, 293T cells were co-transfected with miR-24 mimics and pcDNA3.1-FAF1. As a control, some cells were co-transfected with NC-mimics and pcDNA3.1-FAF1. It was found that miR-24 mimics decreased FAF1 protein levels in a concentration-dependent manner (Fig. 2D and E). In addition, miR-24-ASO, miR-24 mimics and pcDNA3.1-FAF1 were co-transfected into 293T cells. The down-regulation of FAF1 was reversed by miR-24-ASO, but not by NC-ASO (Fig. 2D).

To confirm that miR-24 regulated the FAF1 gene by binding to the two predicted binding sites of ORF, synonymous mutants of these two sites were constructed (Fig. 2B). The 293T cells were co-transfected with miR-24-mimic and pcDNA3.1-FAF1. The mutant FAF1 was not be down-regulated by transfection of miR-24 mimics as much as FAF1/pCDNA3.1 did (Fig. 2F). Taken together, these data suggest that miR-24 can bind and regulate these two response elements in the CDS region of the FAF1 gene.

### miR-24 Regulates Apoptosis in DU-145 Cells by Targeting the FAF1 Gene

**Figure 3:**
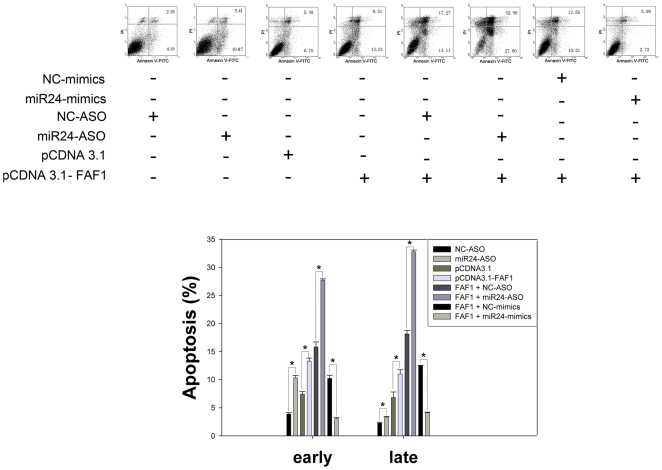
miR-24 regulates apoptosis by targeting FAF. DU-145 cells were treated with the indicated reagents and apoptosis was measured by FACS 48 h after transfection (n = 3±SE, *p<0.01). Not only did down-regulation of miR-24 induce apoptosis, but overexpression of FAF1 also induced apoptosis in DU-145 cells. The percentage of apoptotic cells became much higher when pcDNA3.1-FAF1 and miR-24-ASO were co-transfected. Apoptosis induced by the over-expression of FAF1 could be rescued by the miR-24-mimic. These data show that over-expression of miR-24 can protect DU-145 cells from FAF1-induced apoptosis, and that down-regulation of miR-24 can increase the apoptosis induced by FAF1 in DU-145 cells. Vectors were transfected at the concentration of 100 ng per well; mimics and ASO were transfected at the concentration of 20 nM per well in a 12-well plate.

To test the hypothesis that miR-24 regulates apoptosis in DU-145 cells by targeting the FAF1 gene, we used FACS analysis to investigate the apoptosis of cells transfected with a series of reagents. Over-expression of FAF1 induced apoptosis, as did down-regulation of miR-24. When FAF1 and miR-24-ASO were co-transfected into DU-145 cells, the apoptosis rate was much higher when FAF1 was transfected and miR-24 was down-regulated simultaneously, as compared to results of transfecting either FAF1 or miR-24-ASO alone. Indeed, apoptosis induced by FAF1 could be rescued by the over-expression of miR-24 (Fig. 3). These data indicate that apoptosis induced by down-regulation of miR-24 may act through the regulation of FAF1.

### Mutation of miR-24 Binding Sites in the FAF1 Gene Sensitizes Cells to Apoptosis

**Figure 4:**
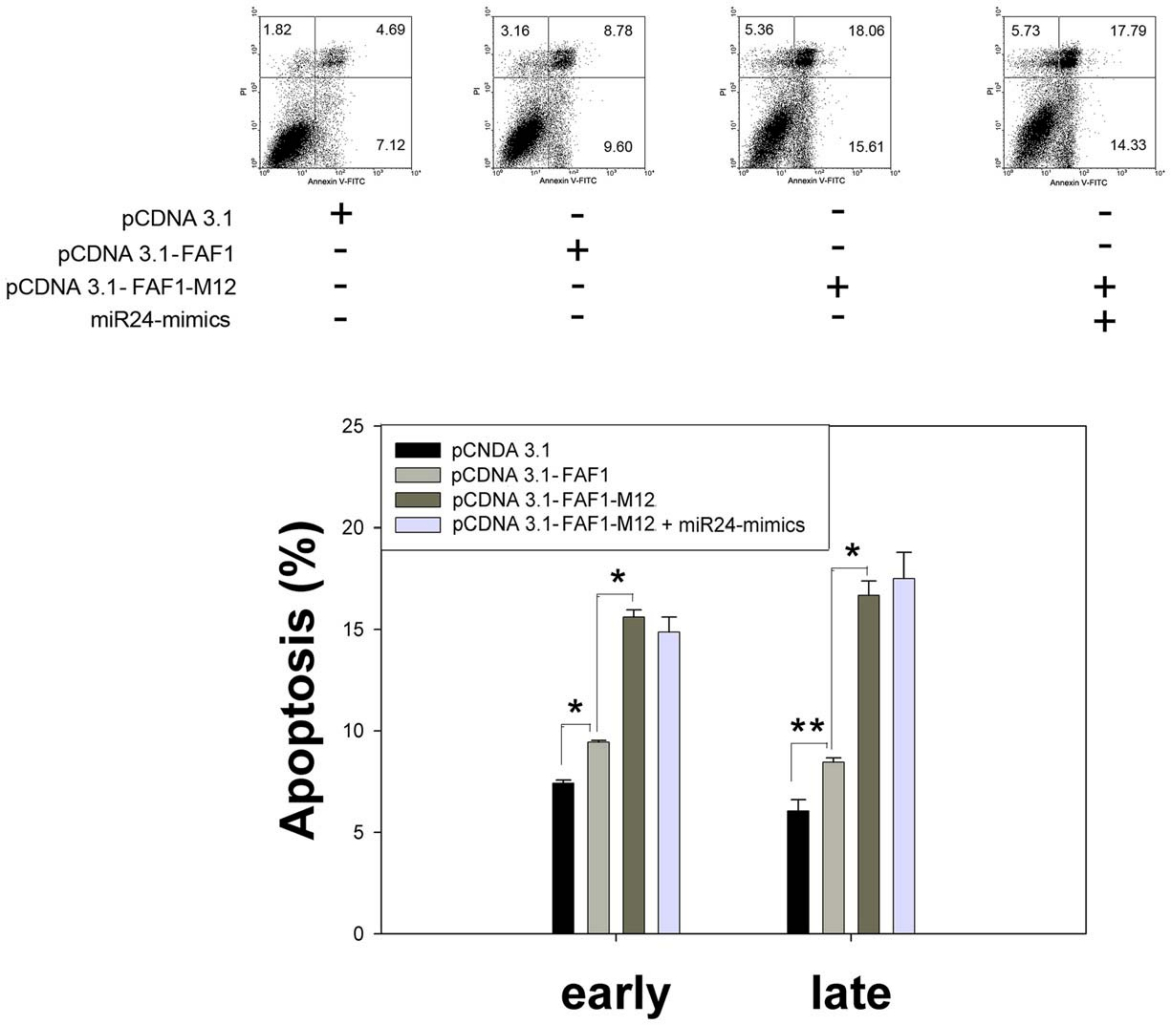
miR-24 regulates apoptosis by binding to the ORF region of the FAF1 gene. DU-145 cells were treated as indicated and apoptosis was measured by FACS 36 h after transfection (n = 3±SE, *p<0.01, **p<0.05). Synonymous mutations at the predicted miR-24 binding sites within FAF1 induced greater levels of apoptosis. Over-expression of miR-24 did not protect DU-145 cells from apoptosis induced by mutant FAF1. Vectors were transfected at a concentration of 100 ng per well; mimics and ASO were transfected at a concentration of 20 nM per well in a 12-well plate.

The binding sites of miR-24 within the FAF1 ORF were mutated in order to further confirm that miR-24 regulates apoptosis in DU-145 cells by targeting the ORF region of the FAF1 gene. The results of this experiment show that DU-145 cells with mutant FAF1 were more susceptible to apoptosis than cells containing the wild-type FAF1 gene. To determine whether the mutant FAF1 could be regulated by miR-24, a mutant FAF1 gene and a miR-24-mimic were co-transfected into DU-145 cells. The over-expression of miR-24 could not rescue the cells from apoptosis induced by mutant FAF1 gene (Fig. 4). These data support our hypothesis that miR-24 regulates apoptosis by binding the ORF region of the FAF1 gene.

### miR-24-ASO-Mediated Down-Regulation of miR-24 Also Induces Apoptosis and Affects Proliferation in HGC-27, MGC-803 and HeLa Cells

**Figure 5:**
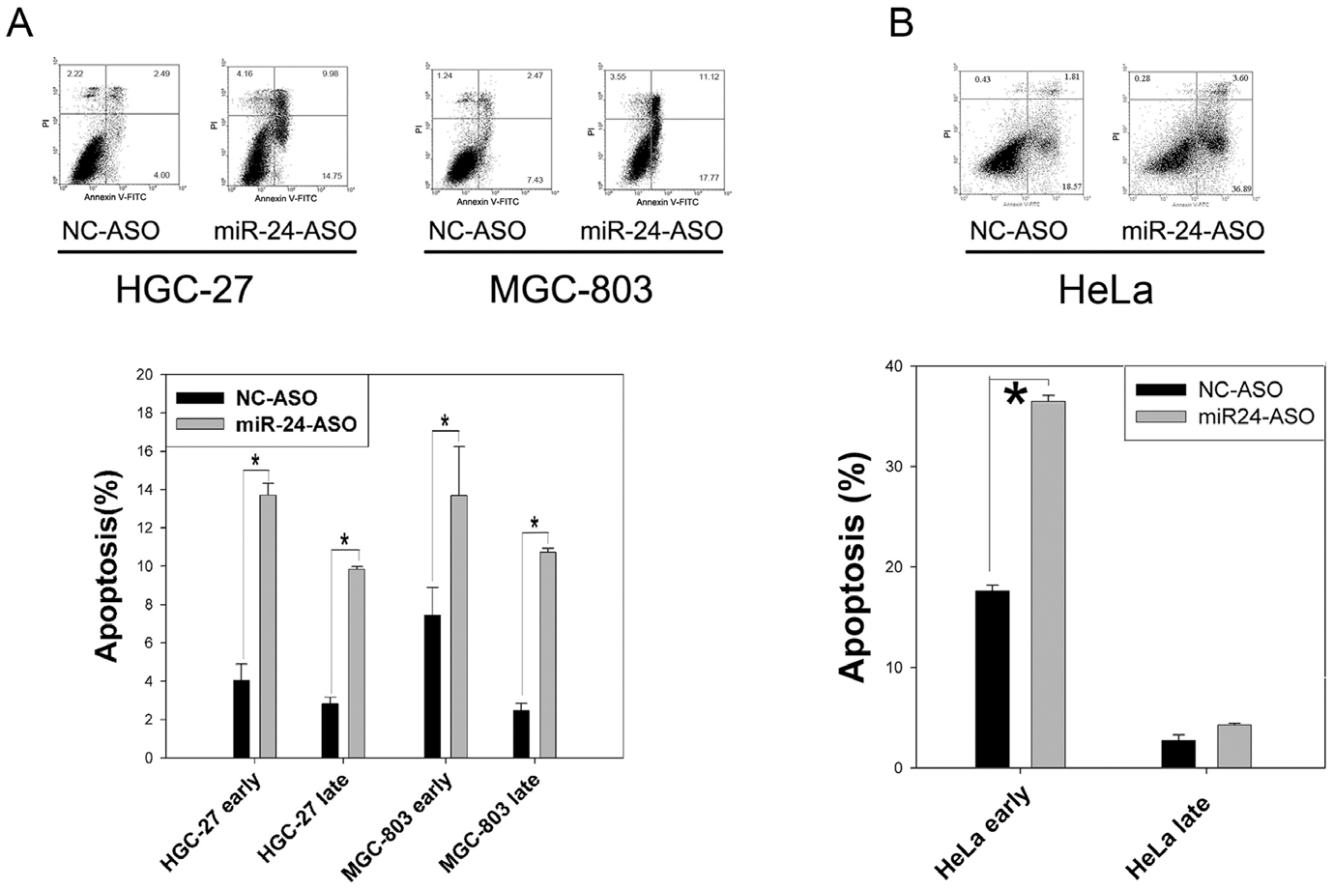
Down-regulation of miR-24 induced apoptosis in HGC-27 and MGC-803 cells, and increased staurosporine sensitivity in HeLa cells. (A) HGC-27 and MGC-803 cells were treated with 20 nM miR-24-ASO or NC-ASO and apoptosis was measured by FACS with Annexin V and propidium iodine staining after 48 h (n = 3±SE; *p<0.01). Down-regulation of miR-24 induced apoptosis in HGC-27 and MGC-803 cells. (B) Apoptosis was induced with 1 µM staurosporine for 2 h after 48 h of transfection using 20 nM miR-24-ASO or NC-ASO (n = 3±SE; *p<0.01). The apoptotic response to staurosporine was increased after transfection with miR-24-ASO.

We next employed the cervical cancer cell line HeLa and two types of human gastric carcinoma cell lines (HGC-27 and MGC-803) to investigate whether miR-24 could regulate apoptosis in other cancer cells. Apoptosis was induced by 20 nM miR-24-ASO without any other induction after 48 hours in HGC-27 and MGC-803 cells (Fig. 5A). In HeLa cells, apoptosis was induced with1 nM staurosporine. The rate of apoptosis in was much higher in cells transfected with 20 nM miR-24-ASO than in cells transfected with NC-ASO (Fig. 5B). The data above suggest that regulation of apoptosis by miR-24 might be a common mechanism in different types of cancer cells.

## Discussion

miRNAs are known to inhibit gene expression by binding to the 3′ UTRs of their target transcripts [34]. Recently, it has been shown that that miRNAs may also modulate gene expression by base-pairing to the CDS region of target mRNAs. For example, the let-7 miRNA directly targets the miRNA-processing enzyme Dicer to its coding sequence, thus establishing a mechanism for a miRNA/Dicer autoregulatory negative feedback loop [12]–[14].

Previous studies have found that down-regulation of FAF1 through RNA interference could enhance the susceptibility to apoptosis [32]. In the present study, we found that miR-24 acts as an endogenous siRNA for FAF1, and apoptosis induced by FAF1 can be rescued by the over-expression of miR-24.

The major finding of this study is that miR-24 targets FAF1 by binding to its CDS region, thereby regulating apoptosis in DU-145 cells. Our study supports an augmented model in which animal miRNAs can regulate mRNAs by binding to targets outside as well as inside the 3′ untranslated region, and extends our understanding of how miR-24 and FAF1 regulate apoptosis in cancer cells.

The function of miRNAs is particularly complicated, and miR-24 may have targets other than FAF1 that are also involved in cell survival. Previous studies have suggested that miR-24 represses the initiation and elongation phases of the translation of mRNA encoding the tumor suppressor p16 (INK4a) [35]. It also has been found that miR-24 is required to repress apoptosis in the developing neural retina [36]. Thus, FAF1 may be one of several distinct targets of miR-24 that contribute to its effect of apoptosis.

DU-145 is the “classical” human prostate cancer cell line, derived from a brain metastasis. DU-145 cells have moderate metastatic potential, are not hormone-sensitive and do not express PSA (prostate specific antigen) [37]–[39]. Hormone-sensitive prostate cancers treated with hormonal therapy typically show good prognosis. However, few effective therapies exist to treat the hormone-insensitive prostate cancers represented by DU-145 [40]. In this study, we found that the over-expression of FAF1 after miR-24 down-regulation induced apoptosis in about 70% of DU-145 cells after 48 h (Fig. 3). This indicates that miR-24 could be a promising therapy target in the treatment of hormone-insensitive prostate cancer.

In addition, miR-24 is one of the most abundant miRNAs in cervical cancer cells [41], and is reportedly up-regulated in solid stomach cancers [42]. Other studies also have shown that FAF1 expression is reduced in gastric carcinomas compared to non-neoplastic tissue, and there was a significant correlation between FAF1 reduction and the content of signet ring cells in gastric carcinomas [43]. These reports suggest that the expression of FAF1 is inversely correlated with the amount of miR-24 in gastric carcinomas. Our study shows that FAF1 is a direct target of miR-24, and that miR-24 can regulate apoptosis in HGC-27, MGC-803 and HeLa cells. These data indicate that regulation of apoptosis through miR-24 suppression of FAF1 may be a common mechanism in several different kinds of cancers.

In conclusion, the present study suggests that the pro-apoptotic factor FAF1 is negatively regulated by miR-24 via two specific target motifs within the FAF1 ORF region. Furthermore, the down-regulation of miR-24 induces apoptosis in DU-145 cells. Our data support an augmented model in which miRNAs can directly target transcripts within their coding region, and suggests that a complete search for the regulatory targets of miRNAs should be expanded to the coding regions of genes. Our findings further reveal miR-24 to be a potential target for gene and drug therapy to treat hormone-insensitive prostate cancer. As similar results were obtained in HGC-27, MGC-803 and HeLa cells, there is also a strong rationale for future investigations of miR-24 as a therapeutic treatment for cervical and gastric cancer.

## Materials and Methods

### Cell Culture and Transfection

Cell lines (293T, HeLa, HGC-27, MGC-803 and DU-145) were obtained from the Cell Bank at the China Academy of Science. Cells were cultured in DMEM, RPMI-1640 or DMEM/F12, all supplemented with 10% fetal bovine serum (FBS), at 37°C in a 5% CO_2_ atmosphere.

Cells at about 70% confluency were transfected using INTERFERin (Polyplus, for transfection with oligonucleotides only) or Lipofectamine 2000 (Invitrogen, for transfection with plasmids) with different concentrations of synthetic miRNA mimics, negative controls (NC-mimics) (Ambion), synthetic LNA-labeled oligo-ribonucleotides, miR-24-ASO and LNA-labeled NC-ASO (TaKaRa) as well as plasmids according to standard protocols. Cells were collected at different time points after transfection for biochemical or biological assays. The oligonucleotide sequences used were:

LNA NC-ASO, 5′ caCTTATCagtcAGACCAtcGT 3′;

LNA 24-ASO, 5′ ctGTTCCTgctgAACTGAgcCA 3′ (small letters indicate the LNA-labeled bases).

### Plasmid Construction

A FAF1 protein expression vector was created by PCR cloning of FAF1 cDNA (NM_007051) between the EcoRI and XhoI sites of pcDNA3.1 (−) (Invitrogen). FAF1 cDNA was amplified from a human cDNA library using the following primers:

5′-GAACTCGAGATTGGCGTCCAACATGGAC-3′ and 5′-GCGCGAATTCTTACTCTTTTGCTTCAAGG-3′. Plasmids containing mutations within the binding sites of miR-24 were constructed with a PCR-based method using the following mutant primers:

FAF1-Mu-B1-FW: 5′-TTAGCTACCTCACGCAAAATTTTATAACCTGGGCTT-3′

FAF1-Mu-B1-RV: 5′-TGCGTGAGGTAGCTAACAATGGATTCAGCACAAAG -3′

FAF1-Mu-B2-FW: 5′-CTCCCTCCGGAACCTAAGGAAGAAAATGCTGAGCCTG-3′

FAF1-Mu-B2-RV: 5′-TTAGGTTCCGGAGGGAGGGCTTGCTCTAAGGACAG-3′

### Luciferase Assay

The miR-24 binding sites were synthesized and cloned into the 3′UTR region of the luciferase gene in the pGL3 luciferase vector (Promega). The obtained construct was co-transfected with PRL-SV40 and miR-24 mimics or NC mimics (Ambion) into 293T cells as previously described. After 48 h, luciferase activity was determined as the average of three independent assays with the Dual-Luciferase Reporter system (Promega).

### Apoptosis Analysis

Each cell line was transfected with synthesized oligonucleotides or vectors. After an additional incubation of 36 or 48 hours, the cells were harvested, stained with propidium iodide and FITC-labeled anti-annexin-V antibody, and analyzed by FACS.

### Cell Proliferation Assay

Cell proliferation assays were performed with a Cell Counting Kit-8 (Dojindo). Cells were plated in 24-well plates in triplicate at about 5×10^4^ cells per well and cultured in the growth medium. At the indicated time points, the numbers of cells per well were measured by the absorbance (450 nm) of reduced WST-8 (2-(2-methoxy-4-nitrophenyl)-3-(4-nitrophenyl)-5-(2, 4- disulfophenyl)-2H-tetrazolium, monosodium salt).

### Protein Extraction and Western Blot

Cells were collected with SDS loading buffer (Sigma). Proteins were separated on SDS-polyacrylamide gels and transferred to a PVDF membrane. The membrane was then blocked with PBST containing 5% milk powder for 1 h at room temperature, followed by hybridization overnight at 4°C in TTBS containing 1% milk powder and primary antibodies. Primary antibodies were detected by a peroxidase-coupled secondary antibody (Sigma) and chemiluminescence (Pierce). The following primary antibodies were used: rabbit anti-FAF (Cell Signaling Technologies) and GAPDH (Abcam).
